# Effects of virtual counseling on the care burden and quality of life of family caregivers for leukemia patients: a randomized controlled trial study

**DOI:** 10.1186/s12912-025-02840-4

**Published:** 2025-02-18

**Authors:** Amir Sadeghi, Vahid Yousofvand, Seyedeh Nayereh Falahan, Sajjad Amiri Bonyad, Behnaz Alafchi

**Affiliations:** 1 Department of Nursing, School of Nursing and Midwifery, Hamadan University of Medical Sciences, Hamadan, Iran; 2 Student Research Committee, School of Nursing and Midwifery, Hamadan University of Medical Sciences, Hamadan, Iran; 3 Student Research Committee, School of Nursing and Midwifery, Shahid Beheshti University of Medical Sciences, Tehran, Iran; 4 Student Research Committee, Hamadan University of Medical Sciences, Hamadan, Iran; 5 School of Public Health, Modeling of Noncommunicable Disease Research Center, Hamadan University of Medical Sciences, Hamadan, Iran

**Keywords:** Virtual counseling, Leukemia, Caregivers, Care burden, Quality of life

## Abstract

**Background:**

Leukemia patients’ caregivers often face care burden and low quality of life. Continuous virtual counseling can help to management these problems. This study examines effects of virtual counseling on the care burden and quality of life of family caregivers for leukemia patients.

**Method:**

The study employed a randomized controlled trial pretest–posttest design with a control group, involving 90 family caregivers of leukemia patients at Iranian oncology clinics in 2021. Two oncology clinics were randomly assigned as experimental (45 participants) and control groups (45 participants). Participants were recruited using a convenience sampling method, adhering to pre-defined inclusion criteria. Data collection was facilitated using Novak and Guest’s Caregiver Burden Inventory and the Caregiver Quality of Life Index-Cancer, administered at baseline, one month, and two months post-intervention. The experimental group engaged in six weeks of continuous virtual counseling, with sessions lasting 45–60 min each week. In contrast, the control group received standard hospital care.

**Results:**

The average ages of the experimental and control groups were 34.29 and 32.33 years, respectively. In the experimental group, 51.1% were men, and 68.88% were spouses of patients. In the control group, 62.2% were women, and 44.45% were spouses of patients. Two months following the intervention, the experimental group demonstrated significant improvement in average scores for both care burden (experimental group: baseline: 90.11 ± 11.34, post-test 1: 73.78 ± 11.58, post-test 2: 52.91 ± 13.57; control group: baseline: 86.38 ± 9.81, post-test 1: 90.93 ± 14.54, post-test 2: 97.40 ± 15.03; a large significant interaction effect for time*group (η^2^ = 0.653, *p* < 0.001), and quality of life (baseline: 65.18 ± 8.36, post-test 1: 73.76 ± 6.53, post-test 2: 89.07 ± 9.43; control group: baseline: 61.82 ± 11.68, post-test 1: 51.96 ± 11.22, post-test 2: 44.24 ± 13.63; a large significant interaction effect for time*group (η^2^ = 0.651, *p* < 0.001).

**Conclusion:**

The findings of this study suggest that virtual counseling can be a positive influence in reducing care burden and improving the quality of life for caregivers of leukemia patients. These results highlight the potential value of incorporating virtual counseling strategies into the caregiving support programs for nurses.

**Trial registration:**

Current controlled trials IRCT20211227053551N7) on February 9, 2025, as well as Retrospectively registered.

## Background

Leukemia, a progressive malignancy, results from the abnormal production of leukocytes, disrupting hematopoiesis and immune function [1]. It accounts for approximately 2.5% of all new cancer cases and 3.1% of cancer-related deaths globally, with age-standardized incidence and mortality rates of 5.4 and 3.3 per 100,000 individuals, respectively [2]. Meanwhile, patients with leukemia often undergo prolonged treatments, such as chemotherapy and radiation, which induce side effects like nausea, fatigue, myalgia, psychological distress, and more [3, 4]. These effects can persist or emerge months to years after treatment, necessitating sustained familial care [5, 6].

In this regard, family caregivers play an important role, providing both physical and emotional support. However, their needs are often overlooked in healthcare systems [3]. Research highlights the significant burdens caregivers face, including psychological distress, physical health issues, financial strain, and social isolation [7]. These challenges, collectively known as care burden, can severely impact caregivers' well-being, leading to diminished quality of life and, in some cases, the need for medical care themselves [3, 8–10].

On the other hand, the caregiving role often escalates caregivers' responsibilities, exposing them to psychological stressors like anxiety, depression, and burnout [11]. Evidence suggests that both leukemia patients and caregivers frequently experience post-traumatic stress symptoms, including hyperarousal, intrusive thoughts, emotional detachment, and depression [12, 13]. Moreover, frequent hospitalizations, follow-ups, and home-based care further elevate caregiver stress, exacerbating guilt and negatively impacting their physical and mental health [12].

Leukemia's impact extends beyond physical caregiving, disrupting social, professional, and daily routines, ultimately affecting caregivers' overall quality of life [14]. For instance, Abbasi et al. (2020) underscored the heightened vulnerability of cancer caregivers to care burden and reduced quality of life, often necessitating additional support [15]. In this regard, caregivers of leukemia patients face significant challenges, including sleep disturbances, anxiety, and diminished quality of life, particularly when supporting patients with active blood cancers. These burdens correlate with unmet support needs, major life disruptions, and lower well-being. Enhanced support through education, consultation, and follow-up is vital for improving outcomes, emphasizing the critical role of nursing professionals [16–18].

Meanwhile, the advent of virtual counseling offers a flexible, cost-effective, and accessible solution to caregivers' challenges, especially during crises like the COVID-19 pandemic [19, 20]. By eliminating commuting and offering adaptable scheduling, it complements traditional counseling methods and expands access to quality healthcare for individuals from diverse backgrounds and locations. As technology evolves, virtual counseling is poised to play an increasingly significant role in healthcare delivery [21–25].

In this context, studies have demonstrated the efficacy of virtual interventions in reducing caregiver burden and improving mental health across various contexts [26, 27]. For instance, Keramitikerman et al. found that peer-support-based online training reduced care burden among leukemia caregivers [28]. Similarly, interventions by Nouri et al., Moradi et al., Juberg et al., and Noei et al. positively impacted caregivers' psychological well-being and reduced long-term care burdens [29–32].

Given these findings, addressing the counseling needs of leukemia caregivers is crucial. Nurses, as key facilitators, can utilize virtual platforms to overcome the challenges of limited time and overcrowded healthcare settings. However, existing research on this topic is limited, highlighting the need for further investigation. Therefore, this study aims to assess the effect of virtual counseling on the care burden and quality of life of family caregivers of leukemia patients.

## Methods

### Design and participants

The study employed a randomized controlled trial pretest–posttest design with a control group. The study sample was recruited from caregivers of leukemia patients who visited the oncology clinics of Shahid Beheshti Hospital and Imam Khomeini Hospital, affiliated with Hamadan University of Medical Sciences, in 2021.

**Fig. 1:**
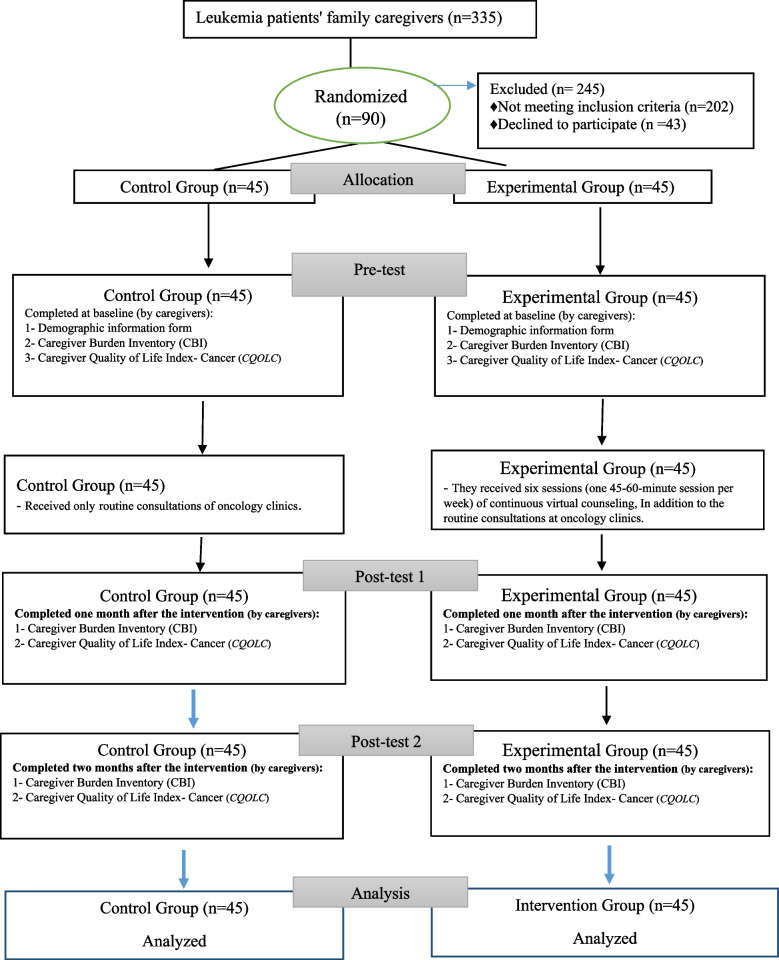
CONSORT diagram

To minimize potential confounding variables arising from variations in clinical practices and resource availability, both oncology clinics in Hamadan were matched based on their general conditions, medical, and nursing services. Subsequently, to ensure unbiased group allocation and reduce the risk of information exchange between groups, a random assignment process was employed using the lottery method (randomization at the group level). Following this randomization, participants who visited the Shahid Beheshti Hospital oncology clinic were assigned to the experimental group, while the caregivers of patients attending the Imam Khomeini (RA) Oncology Clinic were assigned to the control group (Fig. 1). To verify successful randomization and assess baseline equivalence between the experimental and control groups, statistical tests were conducted after collecting the participants' demographic and clinical information, ensuring compliance with all inclusion criteria.

The sample size was determined based on previous study [33], with an 18% increase to account for anticipated attrition. Each group consisted of 45 subjects, calculated using a Type I error (α) of 0.05, a power of 85% (1-β), and an effect size (d) of 0.699. Consequently, 90 caregivers were recruited to participate.

### Inclusion and exclusion criteria

To ensure a homogenous participant pool and minimize extraneous variables, adherence to all inclusion criteria was considered essential in this study.

The inclusion criteria were 1) informed consent for participation, 2) access to a call phone and Internet (at least once a week), 3) ability to speak and listen to answer the questions, 4) At least three months have passed since the diagnosis of leukemia by an oncologist, 5) visiting the oncology clinic at least 2–3 times a month to follow up the treatment of their patients, 6) being over 18, 7) literacy and having the necessary ability to use the cell phone and Internet, 8) not receiving any training based on continuous counseling, improving the quality of life and reducing the care burden of family caregivers, 9) speaking in the Persian language, 10) not suffering from malignant diseases, renal failure, liver disease, chronic obstructive pulmonary disease and neuro-motor disorders, psychiatric and anxiety disorders, and 11) the primary patient care being the responsibility of the participant. In the present study, the definition of "family caregiver" was restricted to immediate family members of the patients. This included spouses, parents, children, and siblings.

Exclusion criteria are 1) death of a caregiver or patient with leukemia during the study, 2) incomplete questionnaires, 3) refusal to continue participation, 4) passing on the caring task to another person, and 5) absence of more than one session in the counseling program.

### Data collection instruments, validity and reliability

In this research, the data collection instruments include personal demographic form, Novak and Guest’s Caregiver Burden Inventory (*CBI*) and Caregiver Quality of Life Index- Cancer (*CQOLC*).

24-items-*CBI* was invented by Novak and Guest in 1989. This inventory includes five subscales: time-dependence burden (5 items), developmental burden (5 items), physical burden (4 items), social burden (5 items) and emotional burden (5 items) [34]. Each item is scored from 0 to 4, with higher scores indicating greater care burden. Thus, time-dependent, developmental, emotional, and social burdens range from 0 to 20, and physical burden ranges from 0 to 16. CBI has three burden stages. 24–47 is mild, 48–71 moderate, 72–95 severe and 96–120 very severe. This inventory is highly reliable, with the Cronbach’s alpha coefficient for the subscales reported from 0.69 to 0.87, and an overall inventory Cronbach’s alpha coefficient is 0.80 [34, 35]. In Iran, Shafizadeh et al. reported the overall internal correlation (Cronbach’s alpha) for the subscales 0.93 and the test–retest reliability coefficient of the intra-cluster correlation index at a two-week interval 0.96 [36]. The internal consistency of the questionnaires was assessed using Cronbach's alpha, which yielded a coefficient of 0.94.

The *CQOLC*, developed by Weitzner et al. in 1997, assesses the impact of cancer on family caregivers' physical, emotional, social, and family functioning. The scale consists of 35 items across five subscales: burden (10 items), disruptiveness (7 items), positive adaptation (7 items), financial concerns (3 items), and other factors (8 items, including sleep disturbance and mental burden). Scores range from 0 to 140, with higher scores indicating better quality of life [37]. Weitzner et al. reported good reliability. Test–retest reliability was 0.95 and internal consistency for the overall scale was 0.90. [38]. The above questionnaire was translatedinto Persian by Khanjari et al. Its face and content validity has been verified. Khanjari et al. reported 4 subscales of the questionnaire including mental/emotional burden, lifestyle disruption, positive adaptation and financial concern: mental/emotional burden containing 14 questions with a maximum score of 56, lifestyle disruption containing 9 questions with a maximum score of 36, positive adaptation containing 8 questions with a maximum score of 32, financial concerns containing 3 questions with a maximum score of 12 and 1 question about “the interest in cooperation in caring for a cancer patient in the family” (item 35), which is not included in any of these subscales and is calculated in the overall quality of life score. 16 questions are scored in reverse. The reliability of this instrument with the internal consistency method (Cronbach's alpha) is 0.89, and the reliability of the subscales includes mental/emotional burden (α = 0.90), lifestyle disorder (α = 0.80), positive adaptation (α = 0.72) and financial concern (α = 0.82) is confirmed [39]. The internal consistency of the questionnaires was assessed using Cronbach's alpha, which yielded a coefficient of 0.93.

Prior to study commencement, informed consent was obtained from all participants in both the experimental and control groups. Participants then completed a demographic information form, the care burden questionnaire, and the quality-of-life questionnaire in a self-administered paper format. One month and two months following the conclusion of the virtual counseling sessions, a researcher (author number 4) coordinated with participants to complete follow-up questionnaires. This follow-up coincided with their scheduled visits to the oncology clinics for treatment services (e.g., chemotherapy) for their patients. The self-administered paper questionnaires, again consisting of the care burden and quality of life measures, took approximately 25–35 min to complete.

To ensure data quality and completeness, all questionnaires were administered under the supervision of another researcher (author number 3). The researcher provided participants with sufficient explanations and addressed any questions that arose. Finally, the completed questionnaires were reviewed for completeness before data entry and analysis by the statistician.

### Intervention

Initially, experimental and control group participants completed a demographic form, the Caregiver Burden Inventory (CBI), and the Caregiver Quality of Life Index-Cancer (CQOLC). For the experimental group, intervention materials were developed and validated by an oncologist to address caregivers' needs, drawing on insights from the aforementioned questionnaires, authoritative texts, and scholarly sources [40–42].

Following development of the virtual counseling intervention materials, a pilot study was conducted at Shahid Beheshti Oncology Clinic to ensure cultural appropriateness and adaptation to the Iranian context. Two researchers (corresponding author and author number 4) reviewed the materials and conducted brief interviews with ten caregivers. Based on this feedback, a virtual counseling program was designed and implemented with the same ten participants. This pilot allowed for identification and rectification of any weaknesses or gaps in the intervention materials.

To assess intervention fidelity in the main study, the lead author delivered a six-week virtual counseling program (offline and online) to the experimental group. Each weekly session lasted 45–60 min. The intervention followed a structured format. First, multimedia content (video clips, audio files, and text) was delivered to participants via mobile phone on a weekly basis. Participants were given 1–3 days to review the materials (offline component) before engaging in an online session with the researcher. The online session began with a brief review of the content, followed by an opportunity for participants to ask questions and clarify any doubts. The researcher then assessed participant comprehension by asking them to explain the content in their own words. Finally, participants were encouraged to ask further questions after the session, and were assured they could contact the researcher through written or voice messages in the virtual space. Throughout the intervention, participants were encouraged to actively participate in discussions and collaboratively search for relevant information.

Content dissemination was tracked using a numerical coding and checkmark system. In cases where participants did not receive content, researchers followed up by phone to identify the reason and, if necessary, resend materials to updated phone numbers. The control group received standard oncology clinic care throughout the study. However, following ethical research principles, all intervention materials were made available to the control group after study completion.

Following the six-week intervention, mobile phone distribution of content ceased. One month later, participants were contacted and asked to complete the Caregiver Burden Inventory (CBI) and the Caregiver Quality of Life Questionnaire (CQOLC) in a self-administered paper format. Subsequently, an online retraining session was held for the experimental group. Two months after the intervention, participants were asked to complete the questionnaires again.

The virtual counseling sessions’ content was as follows:

- Week 1: Familiarization with caregivers with leukemia (definition, cause, side effects, treatment methods, etc.) and responsibility for themselves.
- Week 2: Exercise and physical activity (type, amount, number of times per day, intensity, physical fitness exercises, stretching exercises, etc.)
- Week 3: Education about a healthy diet (getting to know the concept of calories, an introduction to macronutrients and micronutrients, an introduction to food groups and the standard consumption of each food group, ways to increase the duration of following a healthy diet, the correct way to cook food, training how to downsize the plate, reduce the consumption of fast foods, having a daily exercise program to control weight, etc.)
- Week 4: Sleep hygiene and time management (turning the bedroom into a sleep-inducing environment, sticking to a relaxing bedtime routine, avoiding caffeine, alcohol, nicotine, and other chemicals that interfere with sleep, going to bed when feeling tired, setting the body’s biological clock with a fixed sleep schedule, not napping at the end of the day, having a light dinner, drinking fluids in moderation, exercising early in the morning, learning how to plan daily activities, trying to have a fixed daily schedule, etc.)
- Week 5: Stress reduction and positive adaptation (definition, mental, physical, emotional, and behavioral signs of stress, training of internal and external factors that cause stress, correct methods of stress control, managing emotions and unpredictable (emergency) or critical situations, thinking of solutions when facing difficulties (problem-solving), coping with unpredictable and uncertain work situations, intrapersonal adaptation, awareness of the benefits of positivity and creating positive beliefs, learning to recognize one’s strengths, etc.)
- Week 6: improvement of interpersonal and social relations and familiarity with sources of social and financial support and special social networks.

### Ethical considerations

The Ethics Committee in Iran approved this study (ethics code: IR.UMSHA.REC.1400.394, trial registration: IRCT20211227053551N7) on February 9, 2025, as well as Retrospectively registered). This study adhered to the principles outlined in the Declaration of Helsinki. Before the study, the researcher explained the research objectives, assessments, randomization methods, and interventions to the participants at Shahid Beheshti Hospital and Imam Khomeini oncology clinic. We obtained oral and informed consent from all participants to participate and publish the results.

### Data analysis

Data normality was first confirmed using the Kolmogorov–Smirnov and Shapiro–Wilk tests. Descriptive statistics were used to analyze the data and summarize the demographic variables, including frequency, frequency percentage, mean and standard deviation. The data were analyzed using Chi-square tests, Independent Sample t-tests, repeated measures ANOVA, and Bonferroni’s post-hoc test using SPSS 24 at a significance level of *P* < 0.05. Aligned with the study's primary objective of evaluating the intervention's impact on study variables, the effect size fore time*group was determined using partial eta squared (η^2^) derived from a repeated measures analysis of variance (RM ANOVA). The following criteria were employed to interpret the effect size: values below 0.2 indicate a small effect, values between 0.2 and 0.5 indicate a medium effect, and values above 0.5 indicate a large effect.

## Results

The mean age of participants in the experimental group was 34.29 years (SD = 6.69) and 32.33 years (SD = 7.56) in the control group. The experimental group comprised 51.1% males, while the control group comprised 37.8% males (females: 48.9% and 62.2%, respectively). The majority of participants in both groups were spouses of the patients (experimental: 68.88%; control: 44.45%). The level of education was comparable between the groups. In the experimental group, 57.8% of participants had a university degree, while in the control group, the percentage was 73.3%. The most prevalent patient diagnosis in both groups was acute myeloid leukemia. In the experimental group, 60% of participants cared for patients with this diagnosis, while in the control group, the figure was 71.1%. In the analysis of socio-demographic characteristics, no significant between-group difference (experimental and control) was observed (Table 1).

**Table 1:** Socio-demographic characteristics of participants at the baseline (*N* = 90)

Characteristics of participants	Groups		*P*-value
	**Experimental (***N*** = 45)** Mean ± SD or N (%)	**Control (***N*** = 45)** Mean ± SD or N (%)	
**Age (year)**	34.29 ± 6.69	32.33 ± 7.56	0.197^*^
**Salary (million Tomans/month)**	5.93 ± 3.0	5.86 ± 2.94	0.901^*^
**Children**	0.91 ± 1.06	0.76 ± 0.91	0.457^*^
**Care duration (month)**	13.71 ± 7.46	13.64 ± 8.92	0.969^*^
**Care duration during the day (hour)**	8.07 ± 4.12	7.06 ± 3.88	0.582^*^
**Gender (caregiver)**			
Male	23 (51.1%)	17 (37.8%)	0.203^**^
Female	22 (48.9%)	28 (62.2%)	
**Gender (patient)**			
Male	27 (60.0%)	19 (42.2%)	0.092^**^
Female	18 (40.0%)	26 (57.8%)	
**Marital status**			
Single	9 (20.0%)	17 (37.8%)	0.063^**^
Married	36 (80.0%)	28 (62.2%)	
**Residential**			
Urban	39 (86.7%)	42 (93.3%)	0.292^**^
Rural	6 (13.3%)	3 (6.7%)	
**Resident type**			
Personal	23 (51.1%)	30 (66.7%)	0.134^**^
Rental	22 (48.9%)	15 (33.3%)	
**Occupation**			
Employee	37 (82.2%)	35 (77.8%)	0.598^**^
Unemployed	8 (17.8%)	10 (22.2%)	
**Education**			
Non-academic	19 (42.2%)	12 (26.7%)	0.120^**^
Academic	26 (57.8%)	33 (73.3%)	
**Using government assistance**			
Yes	8 (17.8%)	14 (31.1%)	0.141^**^
No	37 (82.2%)	31 (68.9%)	
**Insurance**			
Social security	24 (53.3%)	30 (66.7%)	0.631^**^
Health service	7 (15.6%)	5 (11.1%)	
Armed forces	1 (2.2%)	1 (2.2%)	
Health	13 (28.9%)	9 (20%)	
**Financial dependency**			
Yes	16 (35.6%)	18 (40.0%)	0.664^**^
No	29 (64.4%)	27 (60.0%)	
**Care experience**			
Yes	20 (44.4%)	20 (44.4%)	1.0^**^
No	25 (55.6%)	25 (55.6%)	
**Having alternative**			
Yes	24 (53.3%)	24 (53.3%)	1.0^**^
No	21 (46.7%)	21 (46.7%)	
**Leukemia type**			
AML	27 (60.0%)	32 (71.1%)	0.610^**^
CML	8 (17.8%)	4 (8.9%)	
ALL	8 (17.8%)	7 (15.6%)	
CLL	2 (4.4%)	2 (4.4%)	

^*^^:^^*P*−value derived from independent sample t−test.**:*P*−value derived from chi−square test^

Before the intervention, the caregivers’ care burden was severe (the experimental group was 90.11 (*SD* = 11.34), and the control group was 86.38 (*SD* = 9.81). Baseline analysis revealed no statistically significant difference in mean care burden scores between the experimental and control groups (*p* = 0.205). However, a repeated-measures ANOVA conducted on post-intervention data (experimental group: baseline: 90.11 ± 11.34, one month after the intervention: 73.78 ± 11.58, two months after the intervention: 52.91 ± 13.57; control group: baseline: 86.38 ± 9.81, one month after the intervention: 90.93 ± 14.54, two months after the intervention: 97.40 ± 15.03) showed a large significant interaction effect for time*group (η^2^ = 0.653, *p* < 0.001). This indicates that the change in care burden scores over time differed significantly between the groups. Specifically, the experimental group experienced a greater care burden reduction than the control group (Table 2).

**Table 2:** Mean comparison of "dimensions and total care burden index" of control and intervention groups before, one, and two months after the intervention

Care burden index	Groups		*P*-value
	**Experimental (*n* = 45)** Mean ± SD	**Control (*n* = 45)** Mean ± SD	
**Care burden**			
Before the intervention	90.11 ± 11.34	86.38 ± 9.81	0.205^*^
One month after the intervention	73.78 ± 11.58^1^	90.93 ± 14.54^1^	< 0.001^*^
Two months after the intervention	52.91 ± 13.57^1, 2^	97.40 ± 15.03^1, 2^	< 0.001^*^
***P*-value**	** < 0.001**^†^	** < 0.001**^†^	
	** < 0.001**^¶^		
**Time-dependent care burden**			
Before the intervention	21.27 ± 1.95	20.78 ± 1.68	0.113^*^
One month after the intervention	16.84 ± 3.16^1^	20.93 ± 2.62^1^	< 0.001^*^
Two months after the intervention	11.36 ± 4.06^1, 2^	22.27 ± 2.96^1, 2^	< 0.001^*^
***P*-value**	** < 0.001**^†^	**0.005**^†^	
	** < 0.001**^¶^		
**Developmental care burden**			
Before the intervention	19.47 ± 2.79	18.49 ± 2.99	0.058^*^
One month after the intervention	15.89 ± 3.13^1^	20.38 ± 3.37^1^	0.001^*^
Two months after the intervention	11.98 ± 3.76^1, 2^	20.82 ± 3.73	< 0.001^*^
***P*-value**	** < 0.001**^†^	** < 0.001**^†^	
	** < 0.001**^¶^		
**Physical care burden**			
Before the intervention	15.22 ± 3.20	13.78 ± 3.90	0.870^*^
One month after the intervention	12.04 ± 2.79^1^	14.44 ± 3.67	0.005^*^
Two months after the intervention	8.89 ± 2.68^1, 2^	16.36 ± 3.59^1, 2^	< 0.001^*^
***P*-value**	** < 0.001**^†^	** < 0.001**^†^	
	** < 0.001**^¶^		
**Social care pressure**			
Before the intervention	15.69 ± 4.02	15.82 ± 3.68	0.243^*^
One month after the intervention	13.13 ± 3.04^1^	15.84 ± 5.43	< 0.001^*^
Two months after the intervention	9.38 ± 3.19^1, 2^	17.42 ± 5.19^1, 2^	< 0.001^*^
***P*-value**	** < 0.001**^†^	0.076	
	** < 0.001**^¶^		
**Emotional care burden**			
Before the intervention	18.47 ± 3.56	17.51 ± 4.14	0.098^*^
One month after the intervention	15.87 ± 3.27^1^	19.33 ± 3.53^1^	< 0.001^*^
Two months after the intervention	11.31 ± 2.84^1, 2^	20.53 ± 3.44^1, 2^	< 0.001^*^
***P*-value**	** < 0.001**^†^	** < 0.001**^†^	
	** < 0.001**^¶^		

^*^:*P*-value derived from independent sample t-test. ^†^:*P*-value derived from single repeated measurement. ^¶^:*P*-value derived from overall repeated measurement. ^1^: significantly different with first time point (before intervention). ^2^: Significantly different with second time point (1 month after intervention)

Caregivers’ quality of life was low before the intervention (the experimental group was 65.18 (*SD* = 8.36), and the control group was 61.82 (*SD* = 11.68). Baseline analysis revealed no statistically significant difference in mean quality of life scores between the experimental and control groups (*P* = 0.121). However, a repeated-measures ANOVA conducted on post-intervention data (baseline: 65.18 ± 8.36, one month after the intervention: 73.76 ± 6.53, two months after the intervention: 89.07 ± 9.43; control group: baseline: 61.82 ± 11.68, one month after the intervention: 51.96 ± 11.22, two months after the intervention: 44.24 ± 13.63) showed a large significant interaction effect for time*group (η^2^ = 0.651, *p* < 0.001). This indicates that the change in quality-of-life scores over time differed significantly between the groups. Specifically, the experimental group experienced a greater quality of life increase than the control group (Table 3).

**Table 3:** Mean comparison of "dimensions and total quality of life index" of control and intervention groups before, one, and two months after intervention

Quality of Life Index	Groups		*P*-value
	**Experimental (*N* = 45)** Mean ± SD	**Control (*N* = 45)** Mean ± SD	
**Quality of Life**			
Before the intervention	65.18 ± 8.36	61.82 ± 11.68	0.121^*^
One month after the intervention	73.76 ± 6.53^1^	51.96 ± 11.22^1^	< 0.001^*^
Two months after the intervention	89.07 ± 9.43^1, 2^	44.24 ± 13.63^1, 2^	< 0.001^*^
***P*-value**	** < 0.001**^†^	** < 0.001**^†^	
	** < 0.001**^¶^		
**Mental and physical burden (mental / emotional)**			
Before the intervention	24.44 ± 5.25	22.96 ± 6.46	0.233^*^
One month after the intervention	29.84 ± 4.48^1^	18.58 ± 5.34^1^	< 0.001^*^
Two months after the intervention	38.80 ± 4.63^1, 2^	15.07 ± 7.37^1, 2^	< 0.001^*^
***P*-value**	** < 0.001**^†^	**0.005**^†^	
	** < 0.001**^¶^		
**Lifestyle disruptive**			
Before the intervention	15.87 ± 2.99	14.60 ± 4.03	0.094^*^
One month after the intervention	17.84 ± 2.80^1^	12.73 ± 3.47^1^	< 0.001^*^
Two months after the intervention	22.29 ± 2.68^1, 2^	10.47 ± 4.14^1, 2^	< 0.001^*^
***P*-value**	** < 0.001**^†^	** < 0.001**^†^	
	** < 0.001**^¶^		
**Positive adaptation**			
Before the intervention	15.11 ± 4.12	14.71 ± 5.29	0.690^*^
One month after the intervention	18.67 ± 3.32^1^	14.36 ± 3.98	< 0.001^*^
Two months after the intervention	22.58 ± 4.84^1, 2^	13.60 ± 4.43	< 0.001^*^
***P*-value**	** < 0.001**^†^	0.282^†^	
	** < 0.001**^¶^		
**Financial concerns**			
Before the intervention	7.96 ± 2.63	8.04 ± 2.19	0.862^*^
One month after the intervention	5.87 ± 2.63^1^	4.58 ± 2.60^1^	0.022^*^
Two months after the intervention	4.40 ± 1.78^1, 2^	3.42 ± 2.75^1, 2^	0.049^*^
***P*-value**	** < 0.001**^†^	** < 0.001**^†^	
	** < 0.023**^¶^		

^*^:*P*-value derived from independent sample t-test. ^†^:*P*-value derived from single repeated measurement. ^¶^:*P*-value derived from overall repeated measurement. ^1^: significantly different with first time point (before intervention). ^2^: Significantly different with second time point (1 month after intervention)

## Discussion

This study examined the impact of virtual counseling on the care burden and quality of life of leukemia patients' family caregivers. Consistent with previous research on cancer caregivers [43, 44], participants reported a significant care burden before the intervention. However, this differed from Adili and Dehghani-Arani's (2018) study on breast cancer caregivers, who reported a lower burden [45]. This discrepancy may stem from differences in disease characteristics and treatment duration; leukemia treatment typically lasts longer (13 months) compared to breast cancer (3 months) [46]). The study also identified time-dependent burden as the most significant factor, consistent with leukemia’s cyclical treatment pattern, involving repeated hospitalizations and ongoing home care, which places increasing demands on caregivers.

In this study, the experimental group showed a significant reduction in various care burden dimensions post-intervention, consistent with prior research on cancer care burden interventions [43, 47, 48]. In contrast, the control group experienced an increase in most care burden dimensions, except for the social dimension. These results align with studies demonstrating the effectiveness of support programs in reducing care burden, including telephone training for stroke [49] and Alzheimer's patients [50], as well as nursing interventions for heart failure [51] and stroke caregivers [52]. The lack of intervention, as noted by Ashghali Farahani et al. (2021), often leads to increased care burden after patient discharge [53].

Virtual counseling offers several advantages over traditional in-person interventions, including increased accessibility and reduced costs and time constraints for both caregivers and healthcare professionals [27]. These benefits can empower family caregivers of leukemia patients. Virtual counseling also helps reduce care burden by addressing stress and anxiety [54], while tele-nursing interventions provide caregivers with essential knowledge and skills, fostering self-efficacy and further alleviating care burden [33, 55, 56].

The significant reduction in care burden for the experimental group, compared to the increase in the control group, highlights the effectiveness of the virtual counseling intervention. The program likely provided caregivers with essential knowledge about leukemia, treatment, and self-care strategies, boosting confidence and preparedness. Additionally, the virtual format allowed easier access to healthcare professionals, peer support, and flexibility, helping caregivers manage caregiving responsibilities and emotional stress more effectively. In contrast, the control group experienced increased care burden, likely due to the limited scope of standard oncology care, which did not address caregivers' specific needs, emphasizing the need for supplemental support such as virtual counseling.

On the other hand, the study observed a significant increase in care burden (except social) in the control group, likely due to the lack of virtual counseling support. Without the intervention, caregivers faced challenges alone, leading to increased stress, anxiety, and burden. The intervention group, however, benefited from knowledge about leukemia, treatment, and self-care strategies, boosting their preparedness and confidence. Additionally, the intervention provided stress-management tools, which the control group lacked, contributing to greater emotional strain. The absence of increased social burden in the control group may reflect existing strong social support or the less immediate impact of caregiving on social well-being, which may not change quickly. Further qualitative research could clarify these findings.

This study found low quality of life in both the experimental and control groups before the intervention, consistent with Vashistha et al. and Maziyya et al., who reported similar findings among caregivers of cancer patients [57, 58]. However, these results differ from Lim et al.'s study, which found an average quality of life for caregivers [59], and Clarijs et al.'s report of varying quality of life levels in breast cancer caregivers (average (62.5%), good (35%), and poor (3.5%)) [60]. These discrepancies may be due to differences in study design, sample size, and demographics.

Several factors may account for the better quality of life observed in other studies compared to the present study, including the type of sampling, participant location, specific selection criteria, and the nature of the disease and care methods. In the current study, approximately 55% of participants lacked prior caregiving experience, which likely contributed to the reported low quality of life. Experience in care can help individuals better adapt to the demands of the role and manage the associated stress. Caregiver burden may contribute to low quality of life, as family members often feel responsible for providing compassionate care to leukemia patients, reducing their ability to perform routine activities and self-care. This commitment to caregiving can negatively impact their quality of life. Supporting this, Adili and Dehghani-Arani's study found that care burden accounted for 45% of the variation in quality of life [45].

A significant finding of this study was the significant reduction in financial concerns about quality of life after the intervention, especially in the experimental group. This aligns with Bradley et al.'s observation that caregivers of cancer patients face substantial financial burdens due to intensive care, job loss, high medical expenses, and long-term care [61]. Factors such as job loss, high medical expenses, and long-term care contribute to this financial strain [60]. Financial concerns emerged as a primary issue for caregivers of leukemia patients, indicating a need for additional interventions and support. The COVID-19 pandemic has exacerbated the poor quality of life for caregivers, who often face low wages (an average of 5 million Tomans per month), high rent (48% are tenants), and the substantial costs of patient treatment. Systematic interventions are necessary to support the family caregivers of leukemia patients. Effective measures include workforce support, economic assistance for caregivers, and expert healthcare services. Reducing the burden on family caregivers and improving their quality of life may lead to decreased healthcare resource utilization, enabling caregivers to perform their roles more effectively [62].

Financial burdens significantly affect the mental health, well-being, and quality of life of leukemia caregivers, leading to stress, anxiety, depression, and burnout. These pressures can impair their ability to care for patients and force difficult decisions, contributing to decision fatigue and loss of control. Financial strain can also impact physical health, causing sleep disturbances, appetite changes, and weakened immunity. Caregivers may delay their own healthcare, worsening their condition. Furthermore, financial limitations can lead to social isolation, work performance issues, and family tensions. Policy changes, such as increased insurance coverage, expanded financial aid, flexible work schedules, and tax breaks for caregiving expenses, are crucial to alleviating these burdens. Further research is needed on the long-term effects of financial strain and the effectiveness of such policies.

The comparison of quality-of-life scores across different dimensions before, one month after, and two months after the intervention in the experimental group showed significant improvements. This supports findings by Hu et al. on the effectiveness of educational interventions for stroke caregivers [51] and Meichsner et al. on telephone counseling for dementia caregivers [63]. The COVID-19 pandemic has exacerbated challenges for leukemia caregivers, including financial strain and limited access to healthcare. This study suggests that virtual counseling, particularly by nurses, is an effective solution, as it offers an alternative to in-person visits, which were limited during the pandemic.

This study identified several areas to adapt and improve the virtual counseling intervention for leukemia caregivers. First, addressing financial concerns more directly could enhance the program’s effectiveness. While financial worries were reduced in both groups, the intervention group saw a lesser decrease, suggesting that dedicated modules on managing caregiving costs, financial assistance programs, and budgeting strategies would be beneficial. Additionally, assessing the long-term impact of virtual counseling is important. Follow-up assessments at extended time points (e.g., six months or one year) could help evaluate sustainability and highlight the need for reinforcement sessions or ongoing support.

Further adaptations could include catering to individual needs by offering optional components such as personalized phone counseling or peer support groups. Analyzing participant feedback or conducting focus groups could help optimize the format of the virtual counseling, exploring interactive elements or alternative content delivery methods like webinars or podcasts. Ensuring accessibility for caregivers with varying technological skills or limited device access is crucial, and providing technical support or alternative materials could help. These changes could enhance the program’s ability to meet the diverse needs of caregivers, leading to more sustainable improvements in their quality of life.

## Limitations

The present study has some limitations. This study faced challenges related to its classification and registration as a clinical trial. Initially, the study was not conceptualized as a clinical trial but was later classified as such based on updated guidelines. Due to its retrospective nature, we were unable to register the study prospectively. This lack of pre-registration introduces potential concerns regarding transparency and may limit the ability of readers to fully contextualize the findings. We acknowledge this as a limitation and emphasize our commitment to prospective registration in future studies to uphold scientific rigor and transparency. Most participants had a time limit for using their virtual space, leading the researchers to divide the content into smaller, more manageable parts. The current study's focus on primary caregivers may not capture the full range of family caregiving experiences, potentially introducing selection bias and affecting internal validity. Primary caregivers may experience different levels of burden compared to secondary caregivers. Additionally, the study's findings cannot be generalized to all family members, impacting external validity, as the psycho-emotional burden varies by caregiving role. Future research can include a broader range of family caregivers, such as spouses, adult children, and siblings, to provide a more comprehensive understanding of the care burden. Comparative analyses between primary caregivers and other family members could elucidate how burdens differ across roles. Addressing these limitations would enhance the generalizability of results and offer a more nuanced understanding of caregiving's psychological and emotional impact.

## Conclusion

Cancer affects various aspects of the life of patients’ family caregivers. The study’s results underscore the need for policymakers to provide comprehensive support to family caregivers, both socially and financially. The present study indicates that virtual counseling had a significant positive impact on the care burden and quality of life of leukemia patients’ family caregivers during the COVID-19 pandemic. Therefore, it is recommended to use it as an effective method to reduce the care burden and improve the quality of life of leukemia patients’ caregivers.

## Data Availability

The datasets generated and/or analyzed during the current study are not publicly available due to keeping participants’ information confidential but are available from the corresponding author at reasonable request.
